# Enhanced Positive Emotional Reactivity Undermines Empathy in Behavioral Variant Frontotemporal Dementia

**DOI:** 10.3389/fneur.2018.00402

**Published:** 2018-06-04

**Authors:** Alice Y. Hua, Isabel J. Sible, David C. Perry, Katherine P. Rankin, Joel H. Kramer, Bruce L. Miller, Howard J. Rosen, Virginia E. Sturm

**Affiliations:** ^1^Department of Psychology, University of California, Berkeley, Berkeley, CA, United States; ^2^Department of Neurology, Memory and Aging Center, University of California, San Francisco, San Francisco, CA, United States

**Keywords:** facial electromyography, positive emotion, empathy, dysregulation, emotion recognition, frontotemporal dementia

## Abstract

Behavioral variant frontotemporal dementia (bvFTD) is a neurodegenerative disease characterized by profound changes in emotions and empathy. Although most patients with bvFTD become less sensitive to negative emotional cues, some patients become more sensitive to positive emotional stimuli. We investigated whether dysregulated positive emotions in bvFTD undermine empathy by making it difficult for patients to share (emotional empathy), recognize (cognitive empathy), and respond (real-world empathy) to emotions in others. Fifty-one participants (26 patients with bvFTD and 25 healthy controls) viewed photographs of neutral, positive, negative, and self-conscious emotional faces and then identified the emotions displayed in the photographs. We used facial electromyography to measure automatic, sub-visible activity in two facial muscles during the task: *Zygomaticus major* (*ZM*), which is active during positive emotional reactions (i.e., smiling), and *Corrugator supercilii* (*CS*), which is active during negative emotional reactions (i.e., frowning). Participants rated their baseline positive and negative emotional experience before the task, and informants rated participants' real-world empathic behavior on the Interpersonal Reactivity Index. The majority of participants also underwent structural magnetic resonance imaging. A mixed effects model found a significant diagnosis X trial interaction: patients with bvFTD showed greater *ZM* reactivity to neutral, negative (disgust and surprise), self-conscious (proud), and positive (happy) faces than healthy controls. There was no main effect of diagnosis or diagnosis X trial interaction on *CS* reactivity. Compared to healthy controls, patients with bvFTD had impaired emotion recognition. Multiple regression analyses revealed that greater *ZM* reactivity predicted worse negative emotion recognition and worse real-world empathy. At baseline, positive emotional experience was higher in bvFTD than healthy controls and also predicted worse negative emotion recognition. Voxel-based morphometry analyses found that smaller volume in the thalamus, midcingulate cortex, posterior insula, anterior temporal pole, amygdala, precentral gyrus, and inferior frontal gyrus—structures that support emotion generation, interoception, and emotion regulation—was associated with greater *ZM* reactivity in bvFTD. These findings suggest that dysregulated positive emotional reactivity may relate to reduced empathy in bvFTD by making patients less likely to tune their reactions to the social context and to share, recognize, and respond to others' feelings and needs.

## Introduction

The behavioral variant of frontotemporal dementia (bvFTD) is a neurodegenerative disease characterized by socioemotional decline (1). Patients with bvFTD exhibit dramatic changes in personality and behavior that lead to functional impairment (2). The behavioral symptoms in bvFTD emerge as neurodegeneration selectively targets the frontoinsula, anterior cingulate cortex, thalamus, hypothalamus, amygdala, ventral striatum, and brainstem—brain structures that together form the salience network, a system that supports emotion generation, interoception, and empathy (1–3). Early atrophy in the frontoinsula and anterior cingulate cortex, key salience network hubs, renders certain emotions more vulnerable than others in bvFTD (4, 5). Although specific negative (e.g., disgust) and self-conscious (e.g., embarrassment) emotions are diminished in bvFTD (6–8), certain positive emotions (e.g., happiness) appear to be relatively intact, if not enhanced (9). Some patients with bvFTD exhibit behaviors such as elevated mood, jocularity, and reward-seeking [e.g., pursuit of alcohol and sweets; (10–13)], symptoms that may reflect positive emotion dysregulation (9). Despite this heightened positivity, patients with bvFTD do not exhibit interpersonal warmth (14) or positive emotional responses to social cues that typically promote empathy, compassion, and prosociality (15–17).

Decline in empathy is a core diagnostic feature of bvFTD and is a symptom that has a profound impact on families and caregivers (1, 18, 19). Empathy refers to the ability to feel, understand, and respond to others' emotions (20, 21). As empathy degrades in bvFTD, patients become less sensitive to others' feelings and needs, impairments that erode even longstanding relationships. Numerous studies have shown that emotion recognition and perspective-taking, forms of “cognitive empathy,” are impaired in bvFTD and reflect atrophy in the temporal pole, lateral temporoparietal cortex, and medial prefrontal cortex (22–29). Poor emotion recognition in bvFTD may be due, in part, to impairments in “emotional empathy” (24, 26, 30, 31), an automatic, primitive form of affect-sharing that facilitates emotion recognition (20, 32, 33). During emotional empathy, emotions travel rapidly across individuals via highly conserved visceromotor mirroring systems (20) that include salience network structures such as the frontoinsula, anterior cingulate cortex, midcingulate cortex, and thalamus (34–37). Emotional empathy fosters vicarious affective experience and emotional understanding by allowing individuals to simulate others' internal states. While sharing others' negative emotions can motivate other-oriented behaviors that alleviate suffering, sharing others' positive emotions can create mutual feelings of reward and enjoyment, pleasant feelings that solidify social bonds (38).

In the present study, we examined whether dysregulated positive emotions were associated with empathy impairments in bvFTD. We hypothesized that elevated positive emotional states may make patients with bvFTD less able to feel, recognize, and respond appropriately to others' emotions. Using facial electromyography (EMG), we measured automatic, sub-visible facial muscle reactivity in patients with bvFTD and healthy controls as they viewed photographs of negative, positive, and self-conscious emotional faces. Emotional empathy, which is often assessed by measuring participants' reactions to others' physical or emotional pain (39, 40), can also be measured via facial mimicry (41)—the unconscious, rapid imitation of another's facial expressions. Facial mimicry activates emotion generation systems, enhances emotional experience, and facilitates emotion recognition (42–44). We expected that emotional empathy would be impaired in bvFTD due to atrophy in brain structures that support emotion generation, interoception, and emotion regulation.

Although one approach to quantifying emotional empathy impairments in bvFTD is to measure the extent to which patients' facial reactions are blunted yet context-appropriate (i.e., reduced negative facial reactivity to negative faces and reduced positive facial reactivity to positive faces), another approach is to examine the extent to which patients exhibit facial expressions that are intense but not tuned to the socioemotional context. Whereas previous studies of facial mimicry have focused only on the degree to which an observer's expression matches that of another person (31, 45), here we considered whether emotional empathy impairments in bvFTD may relate to dysregulated positive emotional reactivity (i.e., heightened positive emotional reactions to a wide range of emotional stimuli). We expected that patients with bvFTD who exhibited unmodulated positive emotional reactions to a variety of emotional faces would be worse at recognizing others' emotions and be less responsive to the feelings and needs of people they encounter in their everyday lives.

## Materials and methods

### Participants

Participants included 26 patients with bvFTD recruited through the Memory and Aging Center at the University of California, San Francisco (UCSF) and 25 healthy controls recruited from the community. All participants underwent a detailed clinical interview, neurological examination, functional assessment, and neuropsychological evaluation. Participants completed the neuropsychological testing and diagnostic evaluation in close proximity to the laboratory assessment of emotion (within 4 months for patients and 12 months for healthy controls).

A clinician assessed disease severity using the Clinical Dementia Rating Scale [CDR; (46)]. CDR Total (scores range from 0 to 3) and Sum of the Boxes (CDR-Box; scores range from 0 to 18) scores were calculated for each participant. Higher scores on both CDR measures indicate greater functional impairment. A neuropsychologist assessed cognitive functioning through the Mini Mental State Examination [MMSE; scores range from 0 to 30, with higher scores indicating greater cognitive functioning; (47)] and a comprehensive cognitive battery that included tests of episodic memory (i.e., verbal and visual), executive functioning (e.g., set-shifting, working memory, and fluency), language functioning (e.g., semantic knowledge and confrontational naming), and visuospatial processing.

All patients met consensus research criteria for probable or possible bvFTD (1). The healthy control group underwent an identical neurological and cognitive work-up as the patients and had no history of neurological, psychiatric, or cognitive disorders. The healthy controls had CDR Total and CDR-Box scores of 0 as well as MMSE scores of 26 or above. See Table 1 for demographic information and cognitive test scores for each group.

**Table 1:** Characteristics of participants by group.

**Characteristics**	**Healthy controls**	**bvFTD**	**Statistics and *p*-value**
*N*	25	26	
Age	67.56 (7.63)	63.48 (7.72)	*F*_(1, 49)_ = 3.59, = 0.06
Handedness (% Right)	84	81	χ*^2^*_(1, N = 51)_ = 0.07, = 0.79
Sex (% Female)	68	30.8	χ*^2^*_(1, N = 51)_ = 5.67, = 0.02
Education	17.67 (1.86)	15.73 (2.72)	*F*_(1, 49)_ = 8.49, = 0.005
MMSE	29.00 (1.28)	23.58 (4.25)	*F*_(1, 40)_ = 27.20, < 0.001
CDR-Total	0 (0)	1.17 (0.63)	*F*_(1, 49)_ = 86.19, < 0.001
CDR-Box	0 (0)	6.42 (3.34)	*F*_(1, 49)_ = 92.56, < 0.001
IRI empathic concern	29.50 (4.17)	17.75 (7.85)	*F*_(1, 16)_ = 16.70, < 0.001
IRI perspective-taking	25 (4.35)	14.13 (8.08)	*F*_(1, 16)_ = 13.42, = 0.002
California verbal learning test short form 10 min recall (/9)	^a^	2.5 (2.92)	
Modified trails (correct lines per minute)	43.49 (13.25)	17.40 (12.95)	*F*_(1, 39)_ = 22.37, < 0.001
Modified trails errors	0.22 (0.43)	2.18 (2.65)	*F*_(1, 38)_ = 39.36, < 0.001
Phonemic fluency (# correct in 60 s)	18.69 (3.25)	5.58 (4.03)	*F*_(1, 37)_ = 103.47, < 0.001
Semantic fluency (# correct in 60 s)	25.53 (6.75)	9.58 (6.48)	*F*_(1, 42)_ = 36.20, < 0.001
Design fluency correct (# correct in 60 s)	13.00 (3.20)	5.31 (4.25)	*F*_(1, 41)_ = 40.51, < 0.001
Design fluency repetitions	1.12 (1.22)	5.12 (5.45)	*F*_(1, 41)_ = 8.79, = 0.005
Digits backward	5.59 (1.54)	3.62 (1.50)	*F*_(1, 41)_ = 17.40, < 0.001
Calculations (/5)	4.71 (0.77)	3.48 (1.39)	*F*_(1, 40)_ = 10.91, = 0.002
Benson figure copy (/17)	15.53 (0.99)	14.44 (1.39)	*F*_(1, 38)_ = 2.48, = 0.12
Benson figure copy 10-min recall (/17)	12.87 (2.23)	6.85 (4.61)	*F*_(1, 39)_ = 22.37, < 0.001
Boston naming test spontaneous correct (/15)	14.69 (0.60)	11.69 (3.85)	*F*_(1, 40)_ = 9.42, = 0.003

a *The healthy controls received the California Verbal Learning Test- II (16-word list) instead of the Short-Form. Their performance on the 20-min delay was in the average range expected for individuals their age (M = 12.67, SD = 2.14). bvFTD, behavioral variant frontotemporal dementia; MMSE, Mini-Mental State Examination; CDR Total, Clinical Dementia Rating Total score, and CDR-Box, Clinical Dementia Rating Sum of Boxes; IRI, Interpersonal Reactivity Index. Means (M) and standard deviations (SD) are listed for each group, unless otherwise noted*.

### Procedures

Participants came to the UCSF Center for Psychophysiology and Behavior for a laboratory-based assessment of emotion. All participants or their caregivers, when appropriate, provided informed consent to participate in the study. Participants were seated in a comfortable chair in a well-lit experiment room 1.75 m away from a 21-inch computer monitor. A remotely controlled camera recorded the testing session. The experimental procedures were approved by the UCSF Committee on Human Research.

At the beginning of the testing session, the experimenter used alcohol swabs and mildly abrasive pads to prepare each participant's skin for the application of surface electrodes. Two pairs of 4 mm wide Ag/AgCl electrodes were placed over the left *Zygomaticus major* (*ZM*; cheek) and left *Corrugator supercilii* (*CS*; brow) muscle regions following established facial EMG procedures (48). Whereas *ZM* contraction (which occurs during smiling) is an index of positive emotional reactivity, *CS* contraction (which occurs during frowning) is an index of negative emotional reactivity (30, 32). The left side of the face was chosen due to previous work that has shown that the right hemisphere of the brain plays a predominant role in the production of spontaneous emotional reactions (49). During electrode placement, the experimenter asked participants to smile and frown to verify that the electrodes indeed captured observable changes in *ZM* and *CS* activity. The electrodes were removed and reapplied if they did not capture the expected facial activity or if the inter-electrode impedance levels, which measure the resistance to direct current and reflect noise from the skin surface, were >10 kOhms. The EMG signals were acquired utilizing BIOPAC hardware (one EMG100C amplifier per muscle type) and software (AcqKnowledge version 4.2).

After EMG sensor placement, participants rated their subjective emotional experience of various positive (i.e., amused, compassionate, love or tenderness, and awe) and negative (i.e., afraid, sad, disgusted, and surprised) emotions on a Likert-type scale (0 = not at all to 4 = extremely). These ratings provided us with measures of participants' baseline subjective emotional experience.

### Emotion recognition task

Participants viewed ten photographs of a man displaying various discrete emotional facial expressions. The photographs were selected from the UC Davis Set of Emotion Expressions (50) and included a neutral expression as well as negative (e.g., disgusted, afraid, angry, sad, and surprised), positive (e.g., happy), and self-conscious (e.g., proud, embarrassed, and ashamed) expressions. At the beginning of the task, participants were only instructed to look at the photographs. Each trial was preceded by a 30 s resting baseline period in which participants viewed an “X” on the computer monitor. Participants viewed each photograph for 10 s. All participants viewed the photographs in the same order. After viewing the series of photographs, participants were then shown each photograph again and were asked to identify the emotion of the person in the photograph. They selected their answer from a list of options (i.e., afraid, angry, ashamed, disgusted, embarrassed, happy, neutral, proud, sad, or surprised). All task instructions, questions, and response options were presented visually on the computer monitor and verbally via audio recordings. E-Prime 2.0 software (Psychology Software Tools, Pittsburgh, PA) was used to present the stimuli. One patient with bvFTD did not speak and, thus, did not respond to the multiple choice questions.

### Measures

#### Baseline positive emotional experience

We computed total positive and total negative emotional experience composite scores by summing participants' baseline positive and negative emotion ratings, respectively.

#### Facial EMG reactivity

##### Impedance levels

First, we calculated the mean impedance levels for *ZM* (healthy controls: *M* = 1.89, *SE* = 0.41 and patients: *M* = 1.33, *SE* = 0.21) and *CS* (healthy controls: *M* = 2.10, *SE* = 0.28 and patients: *M* = 1.75, *SE* = 0.17), which were well below the targeted 10 kOhms threshold. The groups did not differ in their mean *ZM, F*_(1, 33)_ = 1.43, *p* = 0.24, or mean *CS, F*_(1, 33)_ = 1.12, *p* = 0.30, inter-electrode impedance levels, which suggested that the electrode placement was adequate and similar for both groups.

##### Data processing and quality checks

Second, the EMG raw signals were filtered offline with a Bandpass Blackman 61 filter (28–500 Hz), integrated, and rectified. 100 ms bins were extracted from the integrated max channels during the last 2 s of the resting baseline and the first 5 s during each photograph. Upon visual inspection, trials in which the raw EMG signals were excessively noisy (e.g., due to poor placement or wire interference) were deleted. The majority of trials for each group were maintained after this first stage of quality checking: 86% of participants (20 patients with bvFTD, 24 healthy controls) had 100% complete trials, and 96% of participants (24 patients with bvFTD, 25 healthy controls) had at least 70% complete trials.

##### Extraneous movement

Third, given that patients with bvFTD occasionally fidget during the testing session, we conducted an additional quality check of the EMG data. A research assistant reviewed the video recordings of each trial of the task and took detailed notes of any extraneous movements that might have impacted the EMG signals (e.g., face-touching, talking, facial twitches, sneezes, and coughs). These notes were then used during another visual inspection of the raw EMG signals. At this stage of the data quality review, 100 ms bins that corresponded to moments of extraneous movements (as determined by the videos) were flagged in the dataset. An index of extraneous movement during each trial was calculated by summing the number of 100 ms bins flagged per trial. Within patients, six bins on average were flagged across all the trials, and within healthy controls, one bin on average was flagged across the trials. The sum of flagged bins across trials was calculated for a total extraneous movement score that was used as a covariate in our analyses.

##### Facial EMG reactivity

**Figure 1:**
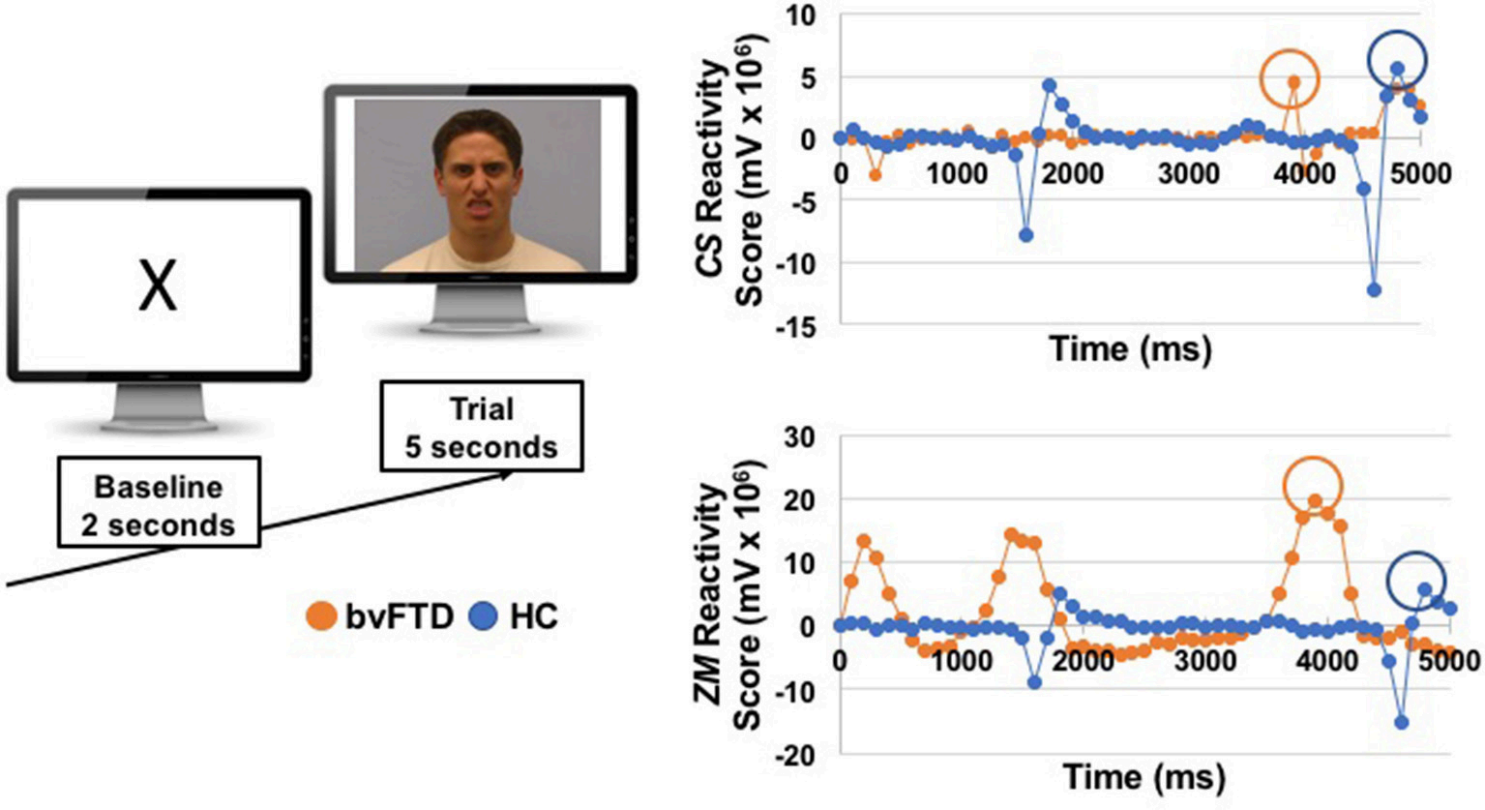
On the left, we show an example of the images that participants viewed during a baseline and a trial. The emotional faces were selected from the UC Davis Set of Emotions Expressions (50); the disgust face from that stimuli set is shown here for illustrative purposes. On the right, we show EMG reactivity scores for one patient with bvFTD and one healthy control (HC) from the disgust trial. Reactivity scores for *zygomaticus major* (*ZM*) and *corrugator supercilli* (*CS*) were calculated by subtracting mean activity during the last 2 s of the baseline from each 100 ms window during the first 5 s of the trial. Peak muscle reactivity was identified for each participant using the maximum change score during the 5 s trial (circled).

We computed two types of scores for each muscle: (1) *peak reactivity scores*, which were calculated for each trial (and used in analyses that examined each trial separately) and (2) *total reactivity scores*, which were calculated across the trials (and used in the correlational behavioral and neuroimaging analyses that examined all trials together). To compute the *peak reactivity scores*, we followed the following procedure. First, baseline *ZM* and *CS* activity were calculated by averaging muscle activity during the last 2 s of the pre-trial baseline period. Second, we calculated the mean activity of *ZM* and *CS* during each 100 ms bin of the first 5 s of the trial. We focused on the first 5 s because previous studies of facial mimicry in patients with socioemotional impairments (e.g., autism and schizophrenia) have used trial windows that range from 1 to 7 s (43, 45, 51, 52), and we wanted to ensure we captured the peak muscle response in a narrow time window while still allowing for variable response times across individuals. Third, we calculated reactivity scores for each trial by subtracting each muscle's mean baseline activity level from its activity during each of the fifty 100 ms bins. Fourth, we identified the peak emotional reactivity score for each muscle to capture the maximum response during each trial. See Figure 1 for an example of one trial. To calculate the *total reactivity scores*, we summed the peak reactivity scores for *ZM* and *CS* across all of the trials, which captured each muscle's total maximal contraction across the task.

To determine whether extraneous movement influenced facial EMG reactivity, we conducted zero order Pearson correlations between total extraneous movement across all trials and total reactivity scores for *ZM* and *CS*. Neither total *ZM* reactivity, *r*_(42)_ = 0.18, 95% CI [−0.12, 0.45], *p* = 0.24, nor total *CS* reactivity, *r*_(43)_ = 0.04, 95% CI [−0.25, 0.33], *p* = 0.78, was associated with total extraneous movement. Thus, extraneous movement did not appear to affect the EMG reactivity scores.

#### Emotion recognition

We calculated a total emotion recognition score, which was the sum of the correctly identified emotions across all ten trials. We also calculated negative (i.e., disgusted, afraid, angry, sad, and surprised), positive (i.e., happy), and self-conscious (i.e., proud, embarrassed, and ashamed) emotion recognition scores by summing the correct emotion recognition responses across each set of relevant trials. Recognition of the neutral face was also examined.

#### Real-world empathy

A subset of participants (7 patients with bvFTD and 9 healthy controls) had informants who completed the Interpersonal Reactivity Index (IRI), a multidimensional measure of real-world empathic behavior, in close proximity to the laboratory-based emotions assessment (within 3 months for patients with bvFTD and 13 months for healthy controls). Given that patients with bvFTD typically lack insight into their behavioral and emotional symptoms, informant reports are a valid way to quantify patients' empathic deficits (23, 24). The IRI is composed of four 7-item subscales (24, 53). Each item was coded on a scale from 1 to 5 (scores for each subscale ranged from 7 to 35). We focused on the empathic concern (a subscale that measures emotional responsiveness to others) and perspective-taking (a subscale that measures the tendency to imagine another person's perspective) subscales because they are established measures of emotional empathy and cognitive empathy, respectively (23).

#### Neuroimaging

Forty-three participants (19 patients with bvFTD and 24 healthy controls) underwent research-quality structural magnetic resonance imaging (MRI) within close proximity to the emotional assessment (within 3 months for patients and 12 months for healthy controls). Structural MRIs were acquired on a 3.0 Tesla Siemens (Siemens, Iselin, NJ) TIM Trio scanner equipped with a 12-channel head coil located at the University of California, San Francisco, Neuroscience Imaging Center using volumetric MPRAGE (160 sagittal slices; slice thickness, 1.0 mm; FOV, 256 × 230 mm^2^; matrix, 256 × 230; voxel size, 1.0 × 1.0 × 1.0 mm^3^; TR, 2,300 ms; TE, 2.98 ms; flip angle, 9°).

After visual inspection, five scans were excluded due to excessive motion or poor scan quality. Thus, 38 scans (23 healthy controls and 15 patients with bvFTD) were included in the neuroimaging analyses. For preprocessing, Statistical Parametric Mapping version 12 default parameters were employed with the light clean-up procedure in the morphological filtering step (http://www.fil.ion.ucl.ac.uk/spm/software/spm12/). Structural T1 images were corrected for bias field, segmented into gray matter, white matter, and cerebrospinal fluid, and spatially normalized into Montreal Neurological Institute (MNI) space (54). Default tissue probability priors (voxel size, 2.0 × 2.0 × 2.0 mm^3^) of the International Consortium for Brain Mapping were used. Segmented images were visually inspected for adequate gray matter segmentation. Segmented images were smoothed with an 8 mm full-width at half-maximum Gaussian kernel.

We used voxel-based morphometry (VBM) to examine the neural correlates of *ZM* and *CS* reactivity in the patients. Statistical maps for VBM were examined at *p* < 0.005, uncorrected. To derive a study-specific error distribution, we ran one thousand permutation analyses to calculate the one-tailed *T*-threshold for correction with multiple comparisons (*p*_FWE_ < 0.05) using vlsm2 (55). This type of permutation analysis uses a resampling approach for significance testing; a test statistic is compared with the null distribution calculated from the present dataset and is an accurate representation of Type 1 error at *p* < 0.05 across the entire brain (56). Images were overlaid with MRIcron (http://people.cas.sc.edu/rorden/mricron/index.html) on a MNI average brain based on the gray and white matter templates used for preprocessing.

## Results

### Participant characteristics

We used analyses of variance (ANOVAs) to compare the groups on age, education, and total extraneous movement. We used a chi-square test to determine whether there were similar proportions of men and women in the patient and control groups. We then used those variables that were significantly different or approached significance (*p* < 0.10) as covariates in our analyses. We also used ANOVAs to compare the functional (CDR-Total and CDR-Box) and cognitive (MMSE and other neuropsychological measures) status between the groups. Means (*M*) and standard errors (*SE*) are presented for each analysis.

The patients with bvFTD and the healthy controls were similar in age, *F*_(1, 49)_ = 3.59, *p* = 0.06. The bvFTD group had a greater proportion of men, χ^2^_(1, N = 51)_ = 5.67, *p* = 0.02, and fewer years of education, *F*_(1, 49)_ = 8.49, *p* = 0.005, than the healthy controls. Thus, we included age, sex, and education in all of our analyses. We also included total extraneous movement, which was higher in the patients with bvFTD than the healthy controls, *F*_(1, 49)_ = 8.40, *p* = 0.006, as an additional covariate in relevant analyses.

Patients were in the mild to moderate range of functioning (as indicated by the CDR) and had impaired cognitive functioning on numerous neuropsychological tests in a battery that included tests of verbal memory (California Verbal Learning Test Short Form 10-min recall) and visual episodic memory [Benson 10-min recall; (57)], confrontational naming [abbreviated Boston Naming Test; (58)]; set-shifting (Modified Trails correct lines per minute); working memory (digits backward); semantic fluency; phonemic fluency; and figural fluency. Demographic information and statistical comparisons for neuropsychological measures are presented in Table 1.

### Baseline positive emotional experience

Analyses of covariance (controlling for age, sex, and education) revealed that patients with bvFTD endorsed significantly greater baseline positive emotional experience than healthy controls, *F*_(1, 36)_ = 11.43, *p* = 0.002 (bvFTD: *M* = 6.09, *SE* = 1.07; healthy controls: *M* = 2.30, *SE* = 0.53). They did not differ significantly from the healthy controls in their baseline negative emotional experience, *F*_(1, 36)_ = 3.52, *p* = 0.07 (bvFTD: *M* = 2.73, *SE* = 1.16; healthy controls: *M* = 0.3, *SE* = 0.13).

### Facial EMG reactivity

We first examined whether the healthy controls exhibited the expected pattern of facial reactions during the task. Consistent with previous facial EMG studies, in the healthy controls peak *ZM* reactivity was greater than peak *CS* reactivity during the positive emotion trial, and peak *CS* reactivity was greater than peak than *ZM* reactivity during the negative emotion trials. During the self-conscious trials, peak *ZM* reactivity was greater than *CS* reactivity, which is consistent with the fact that there is smiling behavior in the target's facial expression for two of the self-conscious trials (i.e., proud and embarrassed). The patients with bvFTD exhibited atypical reactions to numerous trials, as delineated in the analyses that follow. See Table 2 for the means and standard errors of each group's peak muscle reactivity during each trial.

**Table 2:** Facial muscle reactivity by participant group for each facial expression.

		**Healthy Controls**		**bvFTD**	
**Muscle**	**Facial expression**	**Mean**	**Standard error**	**Mean**	**Standard error**
*Zygomaticus major*	Neutral	1.28	0.47	2.68	1.10
	Angry	1.71	1.07	3.43	1.34
	Embarrassed	2.84	2.01	1.25	0.65
	Disgusted	0.82	0.31	5.33	3.63
	Afraid	1.99	1.31	2.72	1.17
	Sad	1.88	0.90	1.85	0.75
	Surprised	1.83	0.72	4.37	2.11
	Proud	1.51	0.63	19.09	16.47
	Ashamed	1.40	0.64	2.69	1.11
	Happy	1.99	0.81	25.55	21.78
	Total	17.67	8.25	76.70	56.53
*Corrugator supercilii*	Neutral	2.11	0.71	2.52	0.78
	Angry	1.70	0.47	3.76	1.37
	Embarrassed	2.23	0.95	2.53	0.71
	Disgusted	2.12	0.62	2.76	0.64
	Afraid	2.38	0.86	5.35	2.19
	Sad	2.99	0.99	6.01	3.37
	Surprised	2.56	0.79	2.30	0.64
	Proud	2.57	1.01	2.68	0.73
	Ashamed	2.18	0.52	12.16	7.96
	Happy	1.79	0.64	4.23	1.22
	Total	23.28	6.71	34.88	8.82

*EMG units are mV × 10^6^*.

We next conducted mixed effects models (with participant as the random effect) to determine whether there were main effects of diagnosis or diagnosis X trial interactions on peak *ZM* and *CS* reactivity (controlling for age, sex, education, and total extraneous movement). These analyses revealed a significant diagnosis X trial interaction on peak *ZM* reactivity, *F*_(1, 487)_ = 4.54, *p* = 0.03, but not on peak *CS* reactivity, *F*_(1, 497)_ = 1.49, *p* = 0.22. Next, we used analyses of covariance (same covariates as above) to decompose the significant diagnosis X trial interaction on *ZM* reactivity. These analyses indicated that patients with bvFTD had significantly greater peak *ZM* reactivity during the neutral, *F*_(5, 43)_ = 5.53, *p* = 0.02, disgusted, *F*_(5, 41)_ = 7.45, *p* = 0.009, surprised, *F*_(5, 41)_ = 4.59, *p* = 0.04, proud, *F*_(5, 42)_ = 4.88, *p* = 0.03, and happy, *F*_(5, 43)_ = 4.76, *p* = 0.04, trials than the healthy controls. To ensure that any demographic or behavioral differences between the groups were not influencing our results, we examined the associations between each muscle's peak reactivity and age, sex, education, and total extraneous movement in our mixed effects models. No significant associations emerged; thus, we concluded that these variables played a minimal role in our results.

To further explore whether sex was impacting our results, we excluded four female healthy controls and conducted a follow-up analysis in a subset of the sample that was sex-matched, χ^2^_(1, N = 45)_ = 3.39, *p* = 0.07. We conducted analyses of covariance (same covariates as above) to examine whether we found a similar pattern of heightened *ZM* reactivity in bvFTD during the neutral, disgusted, surprised, proud, and happy trials. These analyses found that patients with bvFTD continued to have significantly greater peak *ZM* reactivity during the disgusted trial than the healthy controls, *F*_(1, 38)_ = 4.50, *p* = 0.04. Patients with bvFTD also continued to have greater peak *ZM* reactivity during the surprised, *F*_(1, 38)_ = 3.12, *p* = 0.09, proud, *F*_(1, 39)_ = 2.72, *p* = 0.11, and happy, *F*_(1, 39)_ = 2.99, *p* = 0.09, trials compared to healthy controls though these results fell to trend levels due to loss of power in the smaller sample. Given that these analyses found a similar pattern of enhanced *ZM* reactivity in bvFTD during numerous trials, it is unlikely that sex differences between the patients and controls accounted for our results.

Finally, because patients with bvFTD reported elevated positive emotional experience before the task began, we also examined whether higher baseline positive emotional experience was associated with greater *ZM* reactivity. A linear regression (controlling for age, sex, education, and total extraneous movement) across the sample found no association between baseline positive emotional experience and total peak *ZM* reactivity, *r*_(38)_ = 0.23, *p* = 0.17, *t* = 1.77, β = 0.31, *p* = 0.09.

### Emotion recognition

Analyses of covariance (controlling for age, sex, and education) revealed that patients with bvFTD had lower total emotion recognition scores than the healthy controls, *F*_(1, 43)_ = 31.59, *p* < 0.001. Patients with bvFTD were worse than healthy controls at recognizing negative emotional faces, *F*_(1, 44)_ = 38.75, *p* < 0.001 (percent correctly recognized in bvFTD vs. controls: 60 vs. 88% for angry, 36 vs. 92% for disgusted, 32 vs. 68% for afraid, 40 vs. 68% for sad, and 52 vs. 100% for surprised) and the positive emotional face, *F*_(1, 44)_ = 4.33, *p* = 0.04 (84 vs. 100% for happy in bvFTD vs. controls), but their recognition of self-conscious emotional faces was not significantly impaired, *F*_(1, 43)_ = 3.63, *p* = 0.06 (20 vs. 24% for embarrassed, 28 vs. 24% for ashamed, and 58 vs. 100% for proud in bvFTD vs. controls). The patients were also worse at recognizing the neutral face, *F*_(1, 44)_ = 13.66, *p* < 0.001 (48 vs. 92% in bvFTD vs. controls).

### Relationship between positive emotion dysregulation and emotion recognition impairments

We conducted separate linear regressions to examine whether heightened *ZM* reactivity predicted worse emotion recognition across the sample. Linear regressions (controlling for age, sex, education, and total extraneous movement) revealed that greater total *ZM* reactivity across the trials predicted worse recognition of negative emotions, *t* = −2.09, β = −0.39, *p* = 0.04, but not worse recognition of positive, *t* = −0.29, β = −0.05, *p* = 0.77; self-conscious, *t* = −1.46, β = −0.23, *p* = 0.14; or neutral, *t* = 0.62, β = 0.10, *p* = 0.54, faces. Total *CS* reactivity, in contrast, was not associated with recognition of negative, *t* = 0.36, β = 0.07, *p* = 0.72; positive, *t* = 0.56, β = 0.09, *p* = 0.58; self-conscious, *t* = 0.18, β = *0.0*3, *p* = 0.86; or neutral, *t* = −1.41, β = −0.21, *p* = 0.17, faces.

To further investigate the association between positive emotion dysregulation and impaired emotion recognition, we ran a linear regression (controlling for age, sex, and education) to examine whether greater baseline positive emotional experience predicted worse negative emotion recognition. This analysis revealed that greater positive emotional experience at baseline also predicted worse negative emotion recognition, *t* = −3.54, β = −0.58, *p* = 0.001.

### Relationship between positive emotion dysregulation and real-world empathy impairments

**Figure 2:**
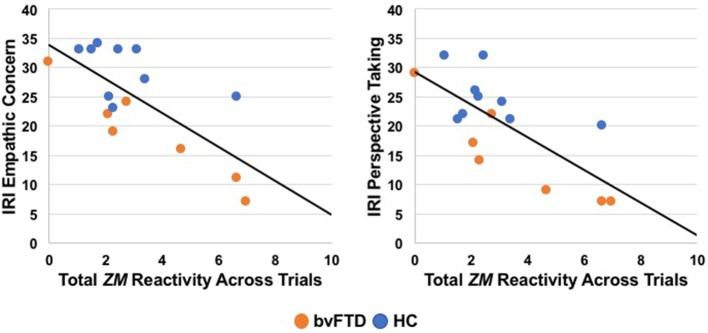
Greater total *zygomaticus major* (*ZM*) peak reactivity across trials correlated with lower empathic concern and perspective-taking on the Interpersonal Reactivity Index (IRI), a measure of real-world empathy that was completed by informants in a subset of participants (7 patients with bvFTD, 9 HC). bvFTD, behavioral variant frontotemporal dementia; HC, healthy controls.

We conducted separate linear regressions across the sample to examine whether heightened *ZM* reactivity predicted worse real-world empathic behavior as measured by the IRI. Linear regressions (controlling for age, sex, education, and total extraneous movement) revealed that elevated *ZM* reactivity was associated with worse real-world empathy. Greater total *ZM* reactivity across the trials predicted lower scores on the empathic concern, *t* = −3.05, β = −0.88, *p* = 0.01, and perspective-taking, *t* = −2.45, β = −0.75, *p* = 0.03, IRI subscales. See Figure 2 for scatterplots. Total *CS* reactivity, however, was not associated with either empathic concern, *t* = −0.40, β = −0.17, *p* = 0.70, or perspective-taking, *t* = −0.25, β = −0.10, *p* = 0.81.

Given the relatively small sample size for the IRI analyses (*n* = 16), we also removed the covariates and conducted zero-order Pearson correlation analyses to confirm that greater total *ZM* reactivity, but not *CS* reactivity, was associated with lower IRI scores. The associations that we detected above remained significant without the covariates: greater total *ZM* reactivity was associated with lower empathic concern, *r*_(16)_ = −0.94, 95% CI [−0.99, −0.54], *p* = 0.004, and perspective-taking, *r*_(16)_ = −0.85, 95% CI [−0.98, −0.12], *p* = 0.03. Similarly, total *CS* reactivity was not associated with either empathic concern, *r*_(16)_ = −0.42, 95% CI [−0.76, 0.10], *p* = 0.11, or perspective-taking, *r*_(16)_ = −0.31, 95% CI [−0.70, 0.22], *p* = 0.24, on the IRI.

To explore whether elevated baseline positive emotional experience also predicted worse real-world empathic behavior, we conducted separate linear regressions (controlling for age, sex, education) in which we tested whether greater subjective positive experience predicted IRI subscale scores. These analyses indicated that baseline positive emotional experience did not predict either empathic concern, *t* = 0.34, β = 0.18, *p* = 0.75, or perspective-taking, *t* = −0.45, β = −0.40, *p* = 0.67, subscale scores.

### Neural correlates of enhanced *Zygomaticus major* reactivity

**Figure 3:**
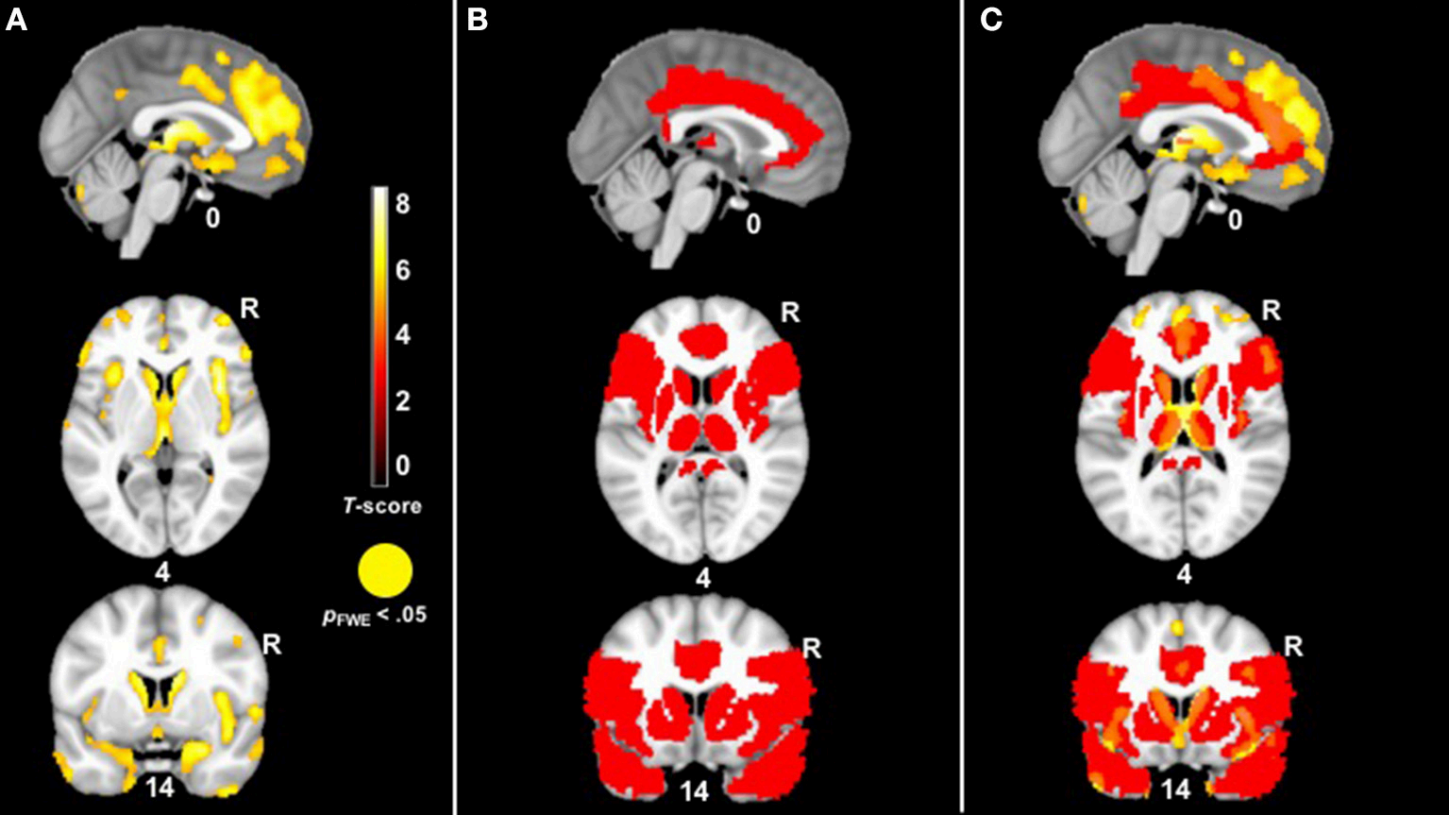
**(A)** *T-*score maps of brain areas in which patients with bvFTD have smaller gray matter volume compared to healthy controls, controlling for age, sex, and total intracranial volume (hot; *p*_FWE_ < 0.05). The patient group had smaller volume in the anterior cingulate, insula, striatum, and amygdala, among other regions. **(B)** The mask for our VBM analysis in red. **(C)** An overlay of both **(A,B)**.

We first conducted a whole-brain analysis in which we compared the patients with bvFTD to the healthy controls (controlling for age, sex, and total intracranial volume) in order to identify regions with significant atrophy. As expected, patients with bvFTD had smaller volume in the insula, anterior cingulate cortex, striatum, and amygdala, among other regions, at the most stringent statistical threshold (*p*_FWE_ < 0.05). See Figure 3.

In our behavioral analyses, the mixed effects models found a significant diagnosis X trial interaction on peak *ZM* reactivity, which indicated that patients with bvFTD had higher *ZM* reactivity than the healthy controls during multiple trials. To capture patients' generalized positive responsivity, we calculated a diagnosis (control = 0, patient = 1) X total *ZM* reactivity (peak reactivity across all trials) interaction term and entered this interaction term as the independent variable in a VBM analysis across the sample. Nuisance covariates included total *ZM* reactivity across all trials, diagnosis, disease severity (CDR-Box), and total intracranial volume (the total volume of gray matter, white matter, and cerebrospinal fluid volume to take into account differences in head size). Because age, sex, education, and total extraneous movement were not significantly associated with peak *ZM* reactivity in our behavioral analyses, we did not include these variables as covariates. In order to offset loss of power incurred by correction for multiple comparisons, we masked our analyses to brain regions that have been implicated in emotion generation, empathy, and facial expression: inferior frontal gyurs (pars triangularis), inferior frontal operculum, insula, cingulate, caudate, putamen, pallidum, thalamus, precentral gyrus, amygdala, and temporal pole. As shown in Figure 3, many of these regions were significantly affected in bvFTD. We also examined whether there were any brain regions in which larger gray matter volume was associated with greater *ZM* reactivity in bvFTD. Furthermore, we conducted parallel analyses for *CS* reactivity and baseline positive emotional experience.

**Figure 4:**
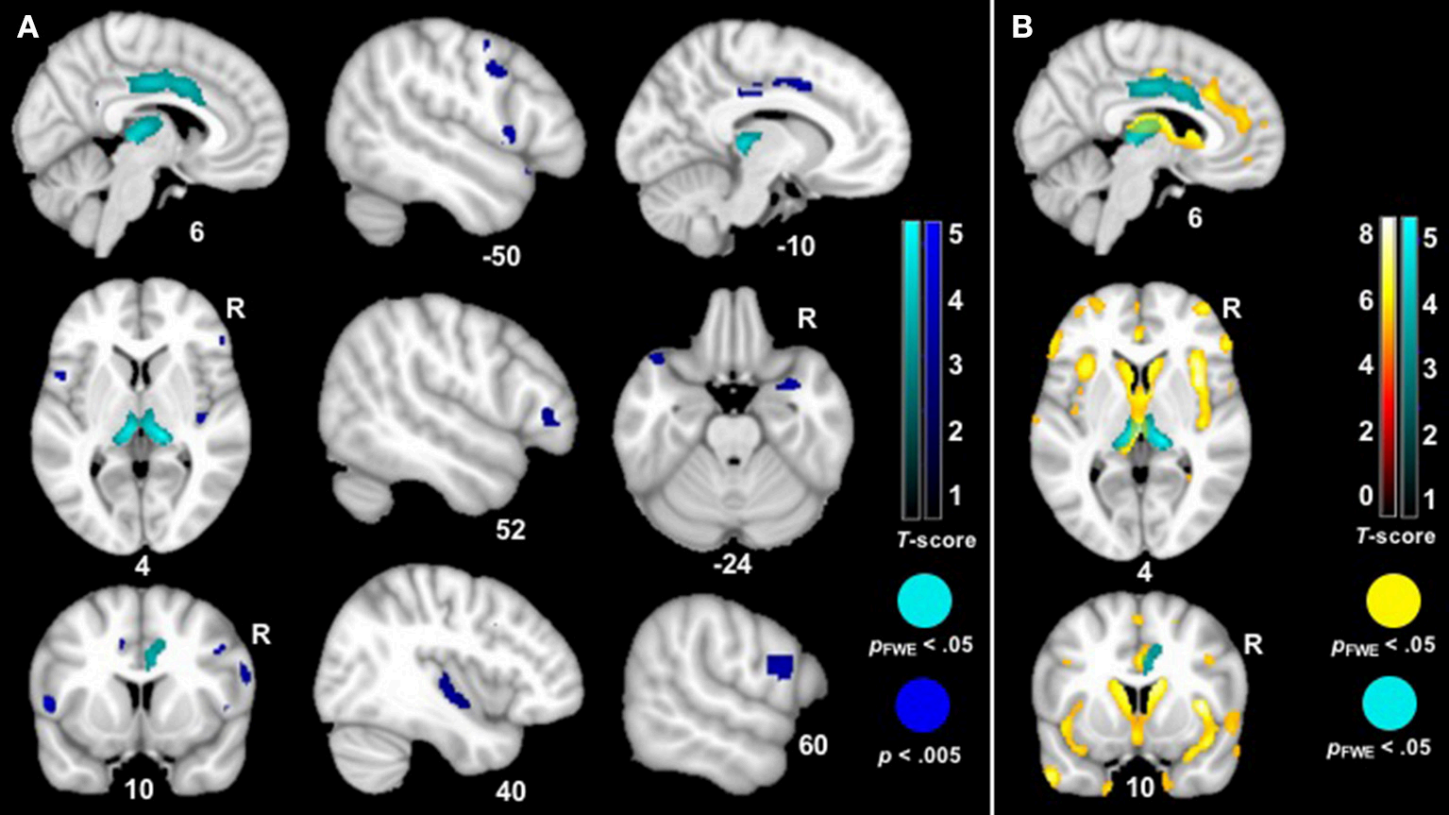
**(A)** *T-*score maps of brain areas in which smaller gray matter volume was associated with greater *zygomaticus major* reactivity in bvFTD. We examined whether there was a *zygomaticus major* X diagnosis interaction on gray matter volume (controlling for the main effects of total *zygomaticus major* peak reactivity across all trials and diagnosis as well as additional nuisance covariates: CDR-Box and total intracranial volume). Smaller volume (Max *T*-score = 5.33) in the bilateral thalamus, bilateral midcingulate cortex, bilateral precentral gyri, left anterior temporal pole, right amygdala, right posterior insula, and bilateral inferior frontal gyrus was associated with greater *zygomaticus major* reactivity across all trials in bvFTD (blue; *p* < 0.005). Clusters in the bilateral thalamus and right midcingulate cortex survived family wise error correction (cyan; *p*_FWE_ < 0.05). Color bars indicate the *T*-scores. **(B)** *T-*score maps of brain areas in which patients with bvFTD have smaller gray matter volume compared to healthy controls (hot; *p*_FWE_ < 0.05) with an overlay of *T-*score maps of brain areas in which smaller gray matter volume was associated with greater *zygomaticus major* reactivity in bvFTD (cyan; *p*_FWE_ < 0.05).

The VBM analysis revealed that smaller volume in the bilateral thalamus and right midcingulate cortex was associated with greater *ZM* reactivity in bvFTD at the most stringent statistical threshold (*p*_FWE_ < 0.05). Smaller volume in the right posterior insula, left anterior temporal pole, bilateral inferior frontal operculum, bilateral precentral gyrus, left midcingulate cortex, and right amygdala was also associated with greater total peak *ZM* reactivity in bvFTD (*p* < 0.005, uncorrected). See Table 3 for *T*-scores and significance levels for all associated regions; Figure 4 displays statistical maps. No brain regions emerged in which larger gray matter volume was associated with greater *ZM* reactivity in bvFTD (*p* < 0.005, uncorrected). There were also no regions associated with *CS* reactivity or baseline positive emotional experience in bvFTD at this threshold.

**Table 3:** Neural correlates of interaction effect between *zygomaticus major* reactivity and the bvFTD diagnosis.

**Anatomical region**	**Cluster volume mm^3^**	**x**	**y**	**z**	**maximum *T*-score**	**Corrected *p*-value**
Right thalamus	5,694	15	−28	4	5.33	0.022^*^
Left thalamus	^†^					
Right midcingulate cortex (anterior and posterior)	5,336	10	−18	34	4.94	0.024^*^
Left precentral gyrus	881	−50	2	38	3.58	0.132
Right posterior insula	776	42	−15	−4	3.61	0.139
Left anterior temporal pole	510	−42	21	−26	3.37	0.175
Right precentral gyrus	500	60	8	20	3.57	0.176
Left midcingulate cortex (anterior)	459	−12	8	40	3.89	0.179
Right amygdala	412	33	4	−24	3.73	0.190
Left midcingulate cortex (posterior)	378	−10	−22	36	3.37	0.196
Left inferior frontal gyrus	365	−52	9	4	4.43	0.197
Right inferior frontal gyrus	230	44	12	38	4.00	0.253
Right inferior frontal gyrus	216	51	34	−3	3.18	0.270
Right posterior cingulate cortex	155	10	−48	26	3.41	0.314

*Smaller volume in bilateral thalami, bilateral midcingulate cortex, bilateral precentral gyri, bilateral inferior frontal operculum, left anterior temporal pole, right amygdala, and right insula was associated with a greater interaction effect between peak zyomaticus major reactivity across all trials and the bvFTD diagnosis when controlling for peak zyomaticus major reactivity across all trials, diagnosis, functional status (CDR-Box), and total intracranial volume. Montreal Neurological Institute coordinates (x, y, z) given for maximum T-score for the cluster (cluster size > 150 mm3). Results are significant at p < 0.005 uncorrected*.

Results considered significant at p < 0.005 uncorrected

* denotes the cluster significant at p_FWE_ < 0.05

† signifies that these regions were included in the cluster above

## Discussion

Our results suggest that atrophy in emotion-relevant brain structures underlies emotional empathy impairment in bvFTD and that a propensity for positive emotional states relates to patients' reduced sensitivity to the feelings and needs of others. Using facial EMG, we found that patients with bvFTD had heightened *ZM* reactivity in response to various types of emotional faces. Patients with bvFTD not only had greater *ZM* reactivity than healthy controls while viewing a positive (i.e., happy) face but also while viewing negative (i.e., disgust and surprise), self-conscious (i.e., proud), and neutral faces. In contrast, patients with bvFTD did not differ from healthy controls in their *CS* reactivity during any trial. In addition, greater total *ZM* reactivity, but not *CS* reactivity, was associated with worse negative emotion recognition and real-world empathy. Furthermore, baseline positive emotional experience was heightened in bvFTD compared to healthy controls, an elevation in positive affect that also predicted worse negative emotion recognition. Taken together, these results suggest that both phasic (i.e., *ZM* reactivity) and tonic (i.e., baseline positive emotional experience) positive emotional responding may be dysregulated—and socially maladaptive—in bvFTD.

### Enhanced positive emotions may undermine negative emotion recognition and real-world empathy

Positive emotions confer numerous benefits such as facilitating approach behavior and fostering social connections (38). Dysregulated positive emotions—positive emotions that are too intense or are context-inappropriate—can be problematic, however, and lead to behavioral symptoms (9, 11, 12). In healthy adults, individuals with lower levels of self-reported emotional empathy exhibit greater *ZM* reactivity to negative (e.g., angry) faces than those with higher emotional empathy (59). In bipolar disorder, a disorder characterized by intermittent periods of mania [a phase defined by positive emotion dysregulation, inappropriate interpersonal boundaries, and risk-taking; (60)] and euthymia (a phase marked by the absence of manic symptoms), empathy may vary across the clinical course. Whereas, during euthymia, individuals with bipolar disorder exhibit typical, or even enhanced, emotion recognition, during mania they have difficulty identifying negative emotions in others (61). In bvFTD, heightened positive emotional reactivity may increase patients' pursuit of rewards and interest in certain types of humor (62, 63) but decrease their sensitivity to feel, know, and respond to others' feelings (15, 24, 64–66).

### Atrophy in emotion-relevant brain structures alters emotions and empathy

The neuroimaging analyses found that smaller gray matter volume in the right midcingulate cortex (anterior and posterior divisions) and bilateral thalamus was associated with greater total *ZM* reactivity in bvFTD. The cluster in the thalamus included the medial pulvinar nucleus and extended into the vicinity of the parvocellular part of the mediodorsal nucleus and ventral posterior lateral nucleus, among others (67). At less stringent statistical thresholds, the left midcingulate cortex, bilateral inferior frontal gyri, bilateral precentral gyri, right amygdala, left temporal pole, and right posterior insula also emerged as regions in which smaller volume was associated with greater total *ZM* reactivity in bvFTD.

The thalamus is a key hub in afferent pathways that receive viscerosensory information and efferent pathways that support skeletomotor control (68, 69, 70, 71). Disruption of thalamocortical loops in bvFTD, therefore, may have a significant effect on emotions and empathy (36, 40, 72–75). The temporal poles, which are critical for appraising the meaning of socioemotional stimuli (76), communicate with the amygdala, a region that is tightly connected with the medial pulvinar nucleus of the thalamus. This system promotes rapid processing of salient visual stimuli (including emotional faces) as well as other incoming sensory information (77, 78). Interoceptive signals from the visceral organs are also relayed to the medial pulvinar, the mediodorsal, and ventral posterior lateral nuclei from brainstem centers (67, 72, 79). This afferent pathway, which has connections to the midcingulate cortex, posterior insula, and the salience network more broadly (69, 70, 72, 80), relays internal signals from the body to the brain and is critical for processing negative, noxious, and painful stimuli ((39, 73, 81). Dysfunction in this system in bvFTD may reduce patients' access to physiological changes that typically accompany shared emotional experiences that motivate empathic and prosocial actions (15).

Although dysfunction in interoceptive pathways may dampen empathy by diminishing negative emotional experience, empathy may also falter as patients are no longer able to suppress positive feeling states, especially in inappropriate contexts. The neuroimaging analyses also indicated that atrophy in regions that produce and regulate facial movements (52) that occur during voluntary facial imitation and spontaneous facial mimicry [e.g., inferior frontal gyrus and primary motor cortex; (52, 82–84)] was associated with greater *ZM* reactivity in bvFTD. Atrophy in the inferior frontal gyrus, a region with a critical role in behavioral and cognitive control (85, 86), may lead to dysregulated emotions in certain contexts. We speculate a combination of diminished reactivity to certain negative emotional cues (6, 7, 87) and enhanced sensitivity to certain positive emotional cues (9, 87) may make patients with bvFTD less able to tune their reactions to the social context and less likely to display empathic responses to others in need.

## Limitations

The present study has several limitations that should be considered. First, we used a variety of emotional faces as stimuli—negative, positive, self-conscious, and neutral—but our ability to assess empathy for positive emotions was limited because we only included a single positive (happy) emotional face. Given that we detected high *ZM* reactivity in bvFTD, an alternate explanation for our results is that patients emotional empathy for positive affective states (e.g., happiness and pride) is enhanced, but we believe this is unlikely. Sharing others' positive emotions fosters close relationships (88), but interpersonal functioning declines significantly in bvFTD (38). Happiness is often the only positive emotion that is assessed in empathy research (45, 49, 51, 52, 59), and it will be important for future studies to investigate how patients with bvFTD respond to other positive emotions, especially those that arise in social contexts [e.g., compassion and affection; (89, 90)]. One previous study found that when the lens on emotion recognition was widened to include patients' identification of numerous positive, negative, and self-conscious emotions, patients with bvFTD demonstrated widespread impairment on all tested emotions, regardless of valence (29). We hypothesize that a similar pattern would emerge for emotional empathy and that, despite enhanced *ZM* reactivity to the happy face in this study, emotional empathy in social situations that typically evoke shared positive feelings would be impaired in bvFTD.

Second, although previous research has found diminished negative emotional reactivity in bvFTD using facial EMG (31), we did not find impairments in *CS* reactivity in the present study. While it is possible that static negative emotional photographs were not intense enough to elicit a measurable *CS* response, previous studies have successfully used photographs to activate the *CS* muscle (49, 52, 91). In addition, the controls in our study displayed the expected *CS* response to the negative faces. We speculate that a proclivity for positivity in bvFTD interferes with context-appropriate empathic responding and, thus, may have obscured *CS* deficits in this sample. Tonic elevations in positive mood or affect may also predispose patients with bvFTD toward positive reactions to emotional—as well as non-emotional—stimuli. If patients were presented with more ecologically valid emotional stimuli that unfolded over time—as emotional events occur in the real world—we hypothesize that patients with bvFTD would have impaired emotional empathy for others' negative as well as positive affective states.

Third, well-established models of empathy have proposed that emotional empathy occurs automatically and promotes cognitive empathy and prosocial actions (33, 92). Consistent with this framework, we examined whether facial EMG reactivity predicted emotion recognition. It is also possible, however, that patients' decline in emotion recognition underlies their facial EMG reactivity alterations and enhanced positive reactions to others' negative emotional states. Patients who have a poor understanding of the social world and others' emotions may be less likely to mirror the emotions of those around them. Future longitudinal studies of bvFTD that investigate the earliest manifestations of empathy disruption would be important for further elucidating how cognitive empathy and emotional empathy interact and decline. Studies of individuals with mutations in *C9ORF72*, a genetic form of bvFTD that targets the medial pulvinar nucleus of the thalamus (93, 94), may help to shed light on this question given the critical role of the thalamus in emotional empathy.

## Conclusion

Emotional empathy is a tuning process during which an individual mirrors and shares the emotions of another person (20, 21, 33). In bvFTD, a disease in which there are profound alterations of emotions and empathy, dysregulation of positive emotions may make patients less able to share, recognize, and respond to the varied affective states of other people.

## Ethics statement

This study was carried out in accordance with adherence to generally accepted practices for experimental research with human subjects. The protocol was approved by the UCSF Committee on Human Research. All subjects or legal guardians gave written informed consent in accordance with the Declaration of Helsinki.

## Author contributions

AH and VS: developed and designed the study; AH and IS: completed data collection; AH and VS: conducted analyses and interpretation of the data; AH, VS, IS, DP, KR, JK, HR, and BM: drafted or revised the manuscript; VS, BM, HR, JK, and KR: acquired financial support for the project leading to this manuscript.

### Conflict of interest statement

KR is a co-author on this manuscript and an editor for this invited issue; thus, KR will not handle any editorial duties for this paper. The other authors declare that the research was conducted in the absence of any commercial or financial relationships that could be construed as a potential conflict of interest.
